# ADHD and Disruptive behavior scores – associations with MAO-A and 5-HTT genes and with platelet MAO-B activity in adolescents

**DOI:** 10.1186/1471-244X-8-28

**Published:** 2008-04-23

**Authors:** Kerstin Malmberg, Hanna-Linn Wargelius, Paul Lichtenstein, Lars Oreland, Jan-Olov Larsson

**Affiliations:** 1 Karolinska Institutet, Department of Woman and Child Health, Child and Adolescent Psychiatric Unit Q3:04, Astrid Lindgren Children's Hospital, Karolinska University Hospital Solna, SE-171 76 Stockholm, Sweden; 2 Uppsala University, Department of Neuroscience, Sweden; 3 Karolinska Institutet, Department of Medical Epidemiology and Biostatistics, Sweden

## Abstract

**Background:**

Pharmacological and genetic studies suggest the importance of the dopaminergic, serotonergic, and noradrenergic systems in the pathogenesis of Attention Deficit Hyperactivity Disorder (ADHD) and Disruptive Behavior Disorder (DBD). We have, in a population-based sample, studied associations between dimensions of the ADHD/DBD phenotype and Monoamine Oxidase B (MAO-B) activity in platelets and polymorphisms in two serotonergic genes: the Monoamine Oxidase A Variable Number of Tandem Repeats (MAO-A VNTR) and the 5-Hydroxytryptamine Transporter gene-Linked Polymorphic Region (5-HTT LPR).

**Methods:**

A population-based sample of twins, with an average age of 16 years, was assessed for ADHD/DBD with a clinical interview; Kiddie Schedule for Affective Disorders and Schizophrenia for School-Age Children-Present and Lifetime Version (K-SADS-PL). Blood was drawn from 247 subjects and analyzed for platelet MAO-B activity and polymorphisms in the MAO-A and 5-HTT genes.

**Results:**

We found an association in girls between low platelet MAO-B activity and symptoms of Oppositional Defiant Disorder (ODD). In girls, there was also an association between the heterozygote long/short 5-HTT LPR genotype and symptoms of conduct disorder. Furthermore the heterozygote 5-HTT LPR genotype in boys was found to be associated with symptoms of Conduct Disorder (CD). In boys, hemizygosity for the short MAO-A VNTR allele was associated with disruptive behavior.

**Conclusion:**

Our study suggests that the serotonin system, in addition to the dopamine system, should be further investigated when studying genetic influences on the development of Disruptive Behavior Disorders.

## Background

Attention Deficit Hyperactivity Disorder (ADHD) and Disruptive Behavior Disorders (DBD), including Conduct Disorder (CD) and Oppositional Defiant Disorder (ODD), are common child and adolescent psychiatric diagnoses. They are disabling and associated with high costs, both for society and in terms of individual suffering. Research regarding these disorders in children and adolescents from the general population is important in order to identify risk factors related to the etiology and prognosis [1]. ADHD affects 3–10% of school aged children [2]. Although the etiology of ADHD is not fully understood, a strong genetic component in the pathogenesis of the disease with an estimated heritability of 60–80% has been reported [1,3]. Consensus estimates suggest that ODD affects 5–10% of children and that 1–5% meet the diagnostic criteria for CD [4]. ADHD is a disorder with two separate underlying symptom dimensions; a hyperactive-impulsive dimension, including excessive activity and impulsive responding, and an inattentive dimension, including difficulties in sustained attention, distractibility, disorganization and lack of task persistence. In the same way DBD includes two dimensions; CD, characterized by a variety of persistent antisocial behaviors including acts of aggression, destruction of property, deceitfulness, theft and violation of commonly adhered to social problems, and ODD, characterized by a sustained pattern of chronic argumentativeness and anger associated with compromised social relations with parents and peers [5].

The symptoms included in the Diagnostic and Statistical Manual of Mental Disorders, 4th edition (DSM-IV), categorical diagnoses of ADHD and DBD can also be studied as dimensions and conceptualized as quantitative variations of behaviors that most individuals show to a greater or lesser degree. There is support for an internal validity of the inattention, hyperactivity/impulsivity, oppositional defiant, and conduct disorder dimensions respectively [6]. Dimensional measures could distinguish different levels within the disorder [7].

Molecular, genetic and pharmacological studies have indicated that the dopaminergic and serotonergic systems play important roles in the development of ADHD [8]. At present, polymorphisms in three dopaminergic loci stand out as the most frequently replicated molecular correlates of ADHD: DRD4, DRD5, and DAT [9,10]. With regard to the serotonergic system, there are recent studies reporting involvement of serotonin in the etiology of ADHD [11]. Serotonergic components are involved in several behavioral traits such as aggression and impulsiveness, which are frequently associated with ADHD [12,13]. Furthermore, Gainetdinov et al [14] found that when administering serotonergic drugs together with methylphenidate to mice lacking the dopamine transporter protein, hyperlocomotor activity was reduced due to increased serotonin levels. Serotonin and dopamine exert regulatory control over each other, suggesting that serotonin, in addition to dopamine, is likely to be linked to ADHD.

Platelet monoamine oxidase (MAO) activity is highly genetically regulated and has repeatedly been associated with temperament [15]. Low platelet MAO-B activity correlates with personality traits such as sensation seeking, impulsivity and monotony avoidance. Platelet MAO-B has also been associated with deviant behavior such as type II alcoholism, which is a risk factor in adult ADHD. MAO-B is considered to be a marker of serotonergic capacity [15] and low activity has previously been associated with ADHD [16]. Two key genes expressing proteins of major importance for serotonergic activity are the genes encoding the serotonin transporter (5-HTT) and the monoamine oxidase A (MAO-A) enzyme. Both of these genes have functional promoter polymorphisms that have been shown to be associated with behavior: the 5-HTT LPR and the MAO-A VNTR [17,18]. A hypothesis which currently gains increasing experimental support is that prenatal serotonin levels are of importance for the development of the central serotonergic system. This hypothesis is supported by molecular genetic [19], pharmacological [20] and brain imaging studies [21].

In the present study, we have tested the hypothesis that a selection of biological markers, related to central serotonergic functioning, are associated with dimensions of the ADHD and DBD phenotype. In a population-based series of adolescent boys and girls, we investigated platelet MAO-B activity and the candidate genes: MAO-A (VNTR) and 5-HTT (LPR).

## Methods

### Study group

**Figure 1:**
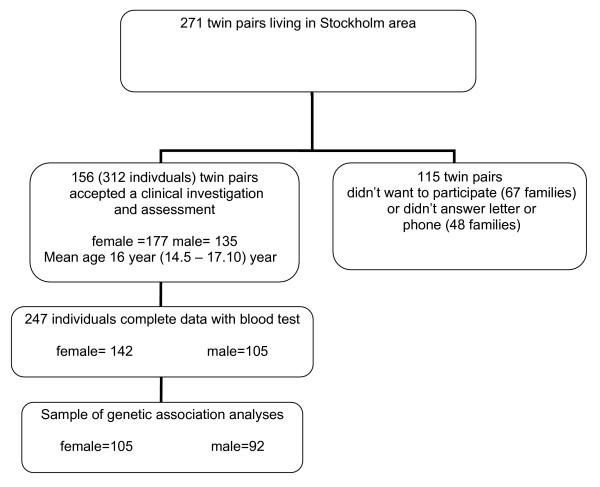
Sample selection procedure.

The subjects were recruited from the population-based TCHAD-study [22], and comprised of twins born from May 1985 to December 1986, living in Stockholm. Of 271 twin pairs, 156 twin pairs i.e. 312 individuals (135 boys and 177 girls), and at least one parent participated in the interview procedure. Mean age was 16 years, ranging from 14.6 to 16.7 years. The sample consisted of 17 pairs of monozygotic boys, 16 pairs of dizygotic boys, 34 pairs of monozygotic girls, 17 pairs of dizygotic girls, and 72 pairs of mixed-sex dizygotic twins. Blood sample was obtained from 247 adolescent individuals (106 boys and 141 girls). Sixty-seven families refused to participate and 48 families did not answer by phone and letters. (Fig 1). Four pairs of twins (8 individuals) were of non-Caucasian origin.

### Clinical interview

The twin pairs and parents were invited to an assessment including a structured clinical interview with Kiddie Schedule for Affective Disorders and Schizophrenia for School-Age Children-Present and Lifetime Version (K-SADS-PL). The K-SADS-PL is a widely used semi structured diagnostic interview designed to assess current and past episodes of psychopathology in children and adolescent according to Diagnostic and Statistical Manual of Mental Disorders, 3rd revised edition (DSM-III-R) and DSM-IV criteria. K-SADS-PL has been shown to be a reliable and valid diagnostic instrument for child and adolescent psychiatric diagnoses [23].

In this study, the present version of the Swedish K-SADS-PL was used to assess the symptoms according to DSM-IV. In the K-SADS-PL interview procedure, the interview was first performed with the child alone and thereafter with at least one parent. The clinical interviewer then summarized the information from the parent (about the child) with the information from the child and classified the symptoms as "not present" (0), "possible" (1) or "certain" (2).

For the purpose of this study, the symptoms included in the criteria for ADHD subtypes, ODD and CD were assessed according to K-SADS-PL and compiled in three ways:

1. Dimensional scales of the symptoms, that is, the summary scores of symptoms included in the criteria for the following diagnoses (scaled 0–2) were calculated: ADHD inattentive type, ADHD hyperactive type, ADHD combined type, ODD, CD and a combined scale for CD and ODD.

2. DSM-IV diagnostic criteria where applied using the information from K-SADS-PL. Each symptom was counted if the item was assessed as "certain" by the interviewer giving the following diagnoses: ADHD inattentive type, ADHD hyperactive type, ADHD combined type, ODD, CD and the combination (CD or ODD) (Table 1).

**Table 1:** Attention-deficit and disruptive behavior disorders derived from Kiddie-SADS – PL interviews in a population-based sample of adolescents

	Girls		Boys		Total	
	
Diagnosis	n/N*	(%)	n/N*	(%)	n/N*	(%)
ADHD inattentive	4/174	2.3	4/134	3.0	8/308	2.6
ADHD hyperactive	2/173	1.2	7/134	5.2	9/307	2.9
ADHD combined	0/173	0.0	1/134	0.8	1/307	0.3
ODD	1/173	0.6	2/133	1.5	3/306	1.0
CD	1/174	0.6	1/133	0.8	2/307	0.7
CD or ODD	2/173	1.2	2/133	1.5	4/306	1.3

	Girls		Boys		Total	
	
High dimensions of phenotype**	n/N*	(%)	n/N*	(%)	n/N*	(%)

ADHD inattentive	29/174	16.7	42/134	31.3	71/308	23.1
ADHD hyperactive	21/173	12.1	25/134	18.7	46/307	15,0
ADHD combined	13/173	7.5	16/134	11.9	29/307	9.5
ODD	23/173	13.3	19/133	14.3	42/306	13.7
CD	9/174	5.2	15/133	11.3	24/307	7.8
CD or ODD	25/173	14.5	25/133	18.8	50/306	16.3

*Number of children fulfilling criteria for: diagnosis or high dimensions of phenotype/total number of children

**Dichotomized symptom scale according to possible and certain symptoms (high/low dimensions of phenotype)

3. The symptoms of the diagnoses described in 1 and 2 were also dichotomized. DSM-IV diagnostic criteria were applied according to the following; the individual child was regarded as having "high score of the ADHD phenotype" if the symptom was assessed as "possible" or "certain" using the same diagnostic criteria as in 2. In order to avoid confusion with clinical diagnoses, we have chosen to use "high/low scores of phenotype" instead of the term "sub threshold" or "probable" diagnosis (Table 1).

To study the validity of the high/low dichotomization we used the Children Global Assessment Scale (CGAS) which is a scale for children very similar with the Global Assessment of Functioning in DSM-IV [24].

### Genotyping and MAO-B activity measurement

Blood samples were obtained from 247 individuals (123 twin pairs and one individual), 106 boys and 141 girls, and genomic DNA was isolated by standard methods. For MAO-B activity measurement, platelet rich plasma was prepared by low-speed centrifugation, 200 × g for 10 minutes. Platelet concentration of the plasma samples were estimated electronically and the plasma was stored at -80°C.

PCR-based genotyping was performed as described in [25] (MAO-A VNTR) and [26] (5-HTT LPR). The MAO-A and 5-HTT PCR products were analyzed by electrophoresis on 2% agarose gels and visualized under UV light by ethidium bromide staining. Genotypes were called in two separate readings.

Because the MAO-A gene is X-linked, only boys were included in the analysis of the MAO-A VNTR. Girls, having two X chromosomes, can be heterozygous and cannot be functionally characterized with certainty because it is not possible to know which of the two alleles is inactivated.

Catalytic activity of platelet MAO-B was analyzed with C^14^-labelled 2-phenylethylamine (β-PEA) as substrate. Before analysis, the samples of platelet rich plasma were thawed and sonicated at 0°C during 5 × 10 seconds with intervals of 5 seconds for lysis of the platelets. 50 μl of the plasma was added to 50 μl of 0.1 mM 14C-β-PEA (0.5 μCi/ml) in 0.1 M sodium phosphate buffer. The reaction mixture was incubated at 37°C for 4 minutes, and the reaction terminated by the addition of 30 μl 1 M HCL. Thereafter, the radioactive aldehyde product formed was extracted by shaking for 30 seconds into 750 μl toluene: ethylacetate (1:1). The samples were then centrifuged at room temperature for 5 minutes at 1000 × g. The organic phase (500 μl), containing the aldehyde product, was pipetted into vials with 8 ml scintillation fluid and the amount of radioactive aldehyde product subsequently quantified by scintillation analysis. Enzyme activity is expressed as nmol of substrate oxidized per 10^10 ^platelets per minute. All samples were analyzed blindly and in duplicate.

### Smoking

There are compounds in cigarette smoke that exert inhibitory effect on MAO activity, however only in quantities exceeding 300 cigarettes per month [27]. Information about cigarette smoking was obtained by asking the subjects, at the time for the blood sampling, whether they had smoked in the past 24 hours (10% boys, 11% girls). This information about smoking was used as a covariate in the statistical analyses of the relationships between behavior and platelet MAO-B activity. The K-SADS-PL inventory contains questions about smoking habits as well. We used smoking more than 2 cigarettes a day as cutoff (11% boys, 12% girls). In order to further explore the possible effects of smoking on our results, subjects with positive smoking information from K-SADS-PL were, for the statistical analysis, combined with the subjects that had been smoking the past 24 hours before the blood test.

### Statistical analyses

Each of the main behavioral symptoms ADHD, ODD and CD, included either as categorical variables (high/low scores of ADHD phenotype) or dimensional scales, were analyzed with respect to their association with MAO-B activity in platelets and genotype of the MAO-A VNTR and the 5-HTT LPR. Separate analyses were performed for boys and girls. For these analyses the general linear model (GLM) in Stata statistical software package was used [28]. Standard errors were adjusted for clustering within twin pairs by increasing the estimated standard errors giving robust estimates of for example p-values. The method is based on the sandwich or Huber/White variance estimator, a method available in Stata 9.0. The descriptive analysis of the data presented in the tables includes the entire sample. However, in the genetic association analyses one of the children in each pair of the MZ twins was randomly excluded from the statistical analyses (n = 51). This is because the MZ twins are supposed to share their genetic background which makes it somewhat uncertain whether the adjustment of standard errors, as described above, is correct for MZ twins.

The dimensional measures of ADHD, ODD and CD problems had skewed distributions. Therefore, for the dimensional measures, non-parametric bootstrap tests were performed using the GLM model in Stata. In the GLM analyses, when calculating the relationships between behavioral problems and 5-HTT genotype, the nominal scales included three variants (long-long, long-short, short-short alleles). This nominal scale was transformed into three index variables and used in the GLM analyses using the long-long variant as the reference variable (omitting this variable). In each of the GLM analyses of platelet MAO-B activity, the information about cigarette smoking, at the time for the blood sampling, whether they had smoked in the past 24 hours, and the smoking information from K-SADS-PL were included as a covariate; smoking (yes/no). For the dimensional ADHD/disruptive behavior scales interactions between sex and polymorphisms in the 5-HTT genotypes were analyzed using GLM. In the same way interactions between sex and platelet MAO-B activity were studied.

Children with missing values for two or more symptoms included in the criteria for each of the diagnoses in this study (the ADHD subtypes, ODD and CD respectively), were excluded from the analyses. If there were missing values for only one of the symptoms in each of the diagnoses this missing value was recorded as "no symptom."

The study was approved by the ethical committee at Karolinska Hospital, Stockholm, Sweden. For all subjects the participating parent and the teenagers gave written informed consent.

## Results

### Prevalence of behavior problems

The observed frequencies of the ADHD phenotype, both for diagnoses and high/low dimensions of phenotype are shown in Table 1. Boys reported more ADHD symptoms than girls. The prevalence of "high scores of ADHD phenotype", inattentive type, was 16.7% in girls and 31.3% in boys; hyperactivity 12.1% in girls and 18.7% in boys; and the combined type 7.5% in girls and 11.9% in boys. For ODD symptoms the prevalence of "high scores of ADHD phenotype" was 13.3% in girls and 14.3% in boys, and for CD symptoms the prevalence was 5.2% in girls and 11.3% in boys. Disruptive behavior (ODD or CD) showed a prevalence of 14.5% in girls and 18.8% in boys.

The CGAS scores were significantly lower (p values from 0.01 to 0.049) in the children with each subtype of the high dimensions of ADHD phenotype (inattentive, hyperactivity and combined types) and DBD (ODD, CD and ODD or CD) compared to children with low dimensions of the phenotype, supporting the validity of the dichotomization.

### Platelet MAO-B activity

Platelet MAO-B activity range was 4.0–19.2 nmol/min/10^10 ^platelets in boys and 6.3–23.7 nmol/min/10^10 ^platelets in girls. Boys had significantly lower mean MAO-B activity than girls (p < 0.001). Low platelet MAO-B activity was associated with higher levels of ODD symptoms and disruptive behavior symptoms in girls but not in boys. This was true for both the DSM-IV dichotomization and for the dimensional analysis (Table 2 and Additional file 1). There were no statistically significant interactions between sex and platelet MAO-B activity related to the dimensional scales of ADHD/disruptive behaviors. Smoking as measured by having smoked the last 24 hours and/or a positive answer about smoking from the K-SADS-PL inventory, did not affect MAO-B activity.

**Table 2:** Dichotomized symptom scale of ADHD/disruptive behavior and platelet MAO-B activity in girls

	MAO-B activity in girls				
Dichotomized symptom scale	High dimension of phenotype Mean(S.D.)	Low dimension of phenotype Mean(S.D.)	n/N*	p^†^	p^‡^

ADHD inattentive	12.57(2.40)	13.31(3.53)	22/140	0.381	0.631
ADHD hyperactive	11.99(2.32)	13.32(3.47)	15/139	0.264	0.215
ADHD combined	11.97(1.95)	13.25(3.45)	8/139	0.646	0.291
ODD	11.50(2.18)	13.44(3.46)	18/140	**0.033**	**0.007**
CD	11.63(1.48)	13.26(3.42)	6/140	0.227	0.167
ODD or CD	11.52(2.07)	13.47(3.48)	20/140	**0.013**	**0.017**

*Number of children with high dimensions of phenotype/total number of children

^† ^Analyses performed using dichotomized symptom scales

^‡ ^Analyses performed using dimensional scales

### MAO-A VNTR in boys

For MAO-A VNTR genotype, 66% of the subjects were hemizygous for the low activity variant and 33% for the high activity allele. Boys with the short MAO-A allele were more likely to show disruptive behavior (p = 0.047) than boys with the long allele (Additional file 2). No significant association was found with regard to the dimensional scales in the sample.

### 5-HTT LPR

For 5-HTT LPR genotype, 41% carried two copies of the long allele, 16% carried two copies of the short, and 43% were heterozygous. The genotype distribution of our sample did not deviate significantly from Hardy-Weinberg equilibrium. 5-HTT genotype in boys was found to be associated with DBD, both using the dimensional scale of CD symptoms (p = 0.006), ODD or CD (p = 0.018) (Table 3), and also in the dichotomized symptom scale (high/low dimensions of phenotype) according to DSM-IV (p = 0.045; Additional file 3)

**Table 3:** Dimensional symptom scales of ADHD and disruptive behavior related to 5-HTT LPR genotype in boys

	5-HTT genotype in boys				
Dimensional symptom scale	SS short/short n = 12 Mean(S.D.)	LS long/short n = 47 Mean(S.D.)	LL long/long n = 45 Mean(S.D.)	p^†^	p^‡^

ADHD inattentive	4.17(3.10)	6.08(4.25)	4.09(3.73)	0.867	0.122
ADHD hyperactive	3.17(3.43)	4.71(4.00)	3.02(2.97)	0.990	0.089
ADHD combined	7.33(6.05)	10.79(7.04)	7.11(6.43)	0.928	0.064
ODD	1.00(1.48)	2.17(2.82)	1.24(2.05)	0.817	0.079
CD	1.08(1.68)	1.49(2.47)	0.42(1.08)	0.328	**0.006**
ODD or CD	2.08(2.64)	3.66(4.90)	1.67(2.86)	0.651	**0.018**

*Total number with the specific genetic marker

^†^SS and LL compared

^‡^LS and LL compared

With regard to 5-HTT genotype in girls, the only statistically significant association was between CD (dimensional scale, p = 0.045) and the long/short genotype (Additional file 4). There were no associations between 5-HTT genotype and dichotomized symptom scales.

## Discussion

In our population-based sample of adolescent twins, we observed significant associations between dimensions of the DBD phenotype and polymorphisms in the candidate genes 5-HTT and MAO-A, as well as with platelet MAO-B activity.

The gender ratio in the present study is somewhat lower than what is usually reported. One explanation for this could be that our sample consists of adolescents that are between childhood and adulthood whereas in other studies subjects are usually children or adults: Meta-analyses of childhood ADHD have shown a male:female ratio of 3:1 in non referred populations [29]. In our study of adolescents we found 1.6:1 for sub threshold diagnosis ADHD combined type. Studies of adult ADHD have shown ratios of 1.6:1 [30] and even 1:1 [31].

Our hypothesis that low platelet MAO-B activity is associated with high dimensions of the ADHD and DBD phenotype was verified only for DBD-symptoms in girls. Acting out anti-social related behaviors is more common and more socially acceptable for boys than for girls and behavioral deviances are thought to be under-diagnosed in girls [32,33]. It is therefore possible that the girls expressing symptoms in the present study had more severe symptoms or more deviant personalities than boys expressing the same behavior. This explanation is in agreement with the discussion by Cederblad et al [34]. Another reason for the relatively lower platelet MAO-B activity in girls with DBD symptoms could be sex-differences in the K-SADS-assessment of behavior based on the reports about aggressive behavior from the parents and teenagers. Alternatively, if the behavioral phenotype is assessed in the same way in boys and girls, without measurement error, there may be real sex-differences in the association between disruptive behavior and serotonergic activity.

Compounds in tobacco smoke inhibit the MAO-B enzyme [35], hence smoking is a confounding factor that must be controlled for when measuring platelet MAO-B activity in humans. There was no difference in MAO activity between subjects that reported smoking and those that did not. Snell et al., 2002 reported that platelet MAO activity was inhibited only after consumption of more than 10 cigarettes per day and we interpret our result that none of our young subjects smoked to that degree [27]. Hence, the present results are not explained by smoking as a confounding factor.

MAO-A VNTR genotype frequencies were in good agreement with those reported by Caspi et al [36]. The 5-HTT genotype frequencies reported here did not differ from those of Gorwood et al [37]. Ethnic origin has an impact on the genotype frequencies in population-based materials, and is a frequent cause of stratification bias. However, in our sample of adolescents from the Swedish twin register, only eight individuals were of non-Caucasian origin.

Traditionally, genes of importance for the serotonergic system, in particular the 5-HTT and MAO-A genes, have been associated with certain traits and behavioral disorders [38]. The MAO-A VNTR has been linked to personality traits such as impulse control and antagonistic behavior in man [39,40]. Hence we hypothesized that MAO-A genotype in boys would be associated with subtypes of ADHD in which aggressive and impulsive behaviors are especially prominent. This was indeed found (Additional file 2). Our findings are in line with those of Lawson et al [41] who found a correlation between the short MAO-A allele and conduct disorder in boys with ADHD. However, there are also studies reporting an association with ADHD related behaviors and the long MAO-A allele [42], as well as studies in which no association were found [8]. Further research is needed to elucidate the direction of gene effects on ADHD related behaviors concerning both the MAO-A and the 5-HTT gene polymorphisms.

Also with regard to the 5-HTT polymorphism, there are conflicting results with studies showing associations between an ADHD phenotype and both the long and the short allele as well as no association at all [10,43]. We found associations between high dimensions of DBD phenotype and 5-HTT LPR heterozygosity in boys and girls. Such molecular heterosis at the 5-HTT gene has been observed in some previous studies where heterozygote subjects have shown a greater effect for binding capacity of the 5-HTT as well as for behavioral traits, i.e. social drinking and depression, than for either of the two homozygotes [44]. Furthermore, in a recent study, central 5-HTT availability was found to be lowest for heterozygous subjects [45]. Heterosis has also been reported for other monoamine receptor genes [44]. The heterosis effect found here points to the importance of conducting analyses on all genotype groups, rather than pooling alleles together as is frequently done in association studies.

We did not genotype the A/G SNP within the 5-HTT LPR [46] which may be a limitation of the study. However, the frequency of this SNP is quite low and the functional importance of it needs to be further studied [47]. Although the strength of the present study is that interviews were carried out by a trained clinician, in contrast to studies that rely on self-completed questionnaires, there is still the possibility of false positives, hence, the positive associations reported here need to be validated in larger sample sets. Finally, when possible, gene-gene interactions and gene-environment interactions are desirable to take into consideration when studying behavioral phenotypes such as ADHD/DBD.

## Conclusion

We found a relationship between low platelet MAO-B activity and ODD, however only in girls. There was, in girls, also an association between the long/short 5-HTT genotype and conduct symptoms. Furthermore, the heterozygote 5-HTT genotype in boys was found to be associated with CD. In boys, homozygosity for the short MAO-A allele was associated with disruptive behavior. Our study suggests that the serotonin system, in addition to the dopamine system should be further investigated when studying genetic influences on the development of Disruptive Behavior Disorders.

## Abbreviations

ADHD: Attention Deficit Hyperactivity Disorder; CD: Conduct Disorder; DAT: Dopamine Transporter; CGAS: Children Global Assessment Scale; DBD: Disruptive Behavior Disorders; DRD4: Dopamine Receptor 4; DRD5: Dopamine Receptor 5; DSM-IV: Diagnostic and Statistical Manual of Mental Disorders, 4th edition; DSM-III-R: Diagnostic and Statistical Manual of Mental Disorders, 3rd revised edition; K-SADS-PL: Kiddie Schedule for Affective Disorders and Schizophrenia for School-Age Children-Present and Lifetime Version; ODD: Oppositional Defiant Disorder; 5-HTT: 5-hydroxytryptamine transporter; MAO-A: Monoamine Oxidase A; VNTR: Variable Number of Tandem Repeats; LPR: gene-Linked Polymorphic Region.

## Competing interests

The authors declare that they have no competing interests.

## Authors' contributions

KM collected clinical data and blood samples, and drafted the manuscript. HW carried out the enzyme measurements and the genotyping, and drafted the manuscript. PL and LO were involved in the conception of the study and in the writing of the manuscript. JOL participated in the design of this study and carried out the statistical analyses and helped draft the manuscript. All authors read and approved the final manuscript.

## Pre-publication history

The pre-publication history for this paper can be accessed here:



## Supplementary Material

Additional file 1 Dichotomized symptom scale of ADHD/disruptive behavior and activity of MAO-B in platelets in boys. Click here for file

Additional file 2 Dichotomized symptom scale of ADHD/disruptive behavior and MAO-A VNTR genotype in boys. Click here for file

Additional file 3 Dichotomized symptom scale of ADHD/disruptive behavior related to 5-HTT LPR genotype in boys. Click here for file

Additional file 4 Dimensional symptom scales of ADHD and disruptive behavior related to genetic markers for 5-HTT LPR genotype in girls. Click here for file
