# Supplementary material for: ADHD and Disruptive behavior scores – associations with MAO-A and 5-HTT genes and with platelet MAO-B activity in adolescents
Source: BMC Psychiatry. 2008 Apr 23;8:28. doi: 10.1186/1471-244X-8-28 (PMC2383890; doi:10.1186/1471-244X-8-28)
Supplement: Additional file 1 — Dichotomized symptom scale of ADHD/disruptive behavior and activity of MAO-B in platelets in boys. [file 1471-244X-8-28-S1.doc]

**Additional file 1 - Dichotomized symptom scale of ADHD/disruptive behavior**

**and activity of MAO-B in platelets in boys**

|  | | | | |
| --- | --- | --- | --- | --- |
| MAO-B activity in boys | | | | |
|  |  |  |  |  |
| Dichotomized symptom scale | High dimension of phenotype  Mean(S.D.) | n/N^*^ | p^†^ | p^‡^ |
|  |  |  |  |  |
| ADHD inattentive | 11.62(2.90) | 32/105 | 0.151 | 0.416 |
| ADHD hyperactive | 11.06(2.58) | 17/105 | 0.878 | 0.548 |
| ADHD combined | 11.31(2.81) | 13/105 | 0.944 | 0.900 |
| ODD | 10.93(2.19) | 17/104 | 0.697 | 0.736 |
| CD | 10.36(2.00) | 11/104 | 0.060 | 0.498 |
| ODD or CD | 10.82(2.20) | 21/104 | 0.330 | 0.608 |
|  |  |  |  |  |

^*^Number of children with high dimensions of phenotype/total number of children

^†^ Analyses performed using dichotomized symptom scales

^‡^ Analyses performed using dimensional scales
