# Supplementary material for: ADHD and Disruptive behavior scores – associations with MAO-A and 5-HTT genes and with platelet MAO-B activity in adolescents
Source: BMC Psychiatry. 2008 Apr 23;8:28. doi: 10.1186/1471-244X-8-28 (PMC2383890; doi:10.1186/1471-244X-8-28)
Supplement: Additional file 2 — Dichotomized symptom scale of ADHD/disruptive behavior and MAO-A VNTR genotype in boys. [file 1471-244X-8-28-S2.doc]

**Additional file 2 - Dichotomized symptom scale of ADHD/disruptive**

**behavior and MAO-A VNTR genotype in boys**

|  |  | |  |  |
| --- | --- | --- | --- | --- |
|  | MAO-A genotype | |  |  |
|  |  |  |  |  |
| High dimensions of phenotype** | Short  3 repeat | Long  4 repeat | p^†^ | p^‡^ |
|  | n/N^*^ | n/N^*^ |  |  |
|  |  |  |  |  |
| ADHD inattentive | 21/71 | 12/35 | 0.786 | 0.620 |
| ADHD hyperactive | 9/70 | 8/35 | 0.444 | 0.402 |
| ADHD combined | 7/70 | 6/35 | 0.621 | 0.351 |
| ODD | 8/70 | 9/35 | 0.169 | 0.501 |
| CD | 5/70 | 6/35 | 0.298 | 0.389 |
| ODD or CD | 9/70 | 12/35 | **0.047** | 0.431 |
|  |  |  |  |  |

^*^Number of boys with high dimensions of phenotype/total number with the

specific genetic marker

**Dichotomized symptom scale according to possible and certain symptoms

(high/low dimensions of phenotype)

^†^Analyses performed using dichotomized symptom scale

^‡^Analyses performed using dimensional scale
