# Supplementary material for: ADHD and Disruptive behavior scores – associations with MAO-A and 5-HTT genes and with platelet MAO-B activity in adolescents
Source: BMC Psychiatry. 2008 Apr 23;8:28. doi: 10.1186/1471-244X-8-28 (PMC2383890; doi:10.1186/1471-244X-8-28)
Supplement: Additional file 3 — Dichotomized symptom scale of ADHD/disruptive behavior related to 5-HTT LPR genotype in boys. [file 1471-244X-8-28-S3.doc]

**Additional file 3 - Dichotomized symptom scale of ADHD/disruptive behavior**

**related to 5-HTT LPR genotype in boys**

|  |  | | |  |  |
| --- | --- | --- | --- | --- | --- |
|  | 5-HTT genotype in boys | | |  |  |
|  |  |  |  |  |  |
| High dimensions of phenotype** | SS  short/short | LS  long/short | LL  long/long | p^†^ | p^‡^ |
|  | n/N^*^ | n/N^*^ | n/N^*^ |  |  |
|  |  |  |  |  |  |
| ADHD inattentive | 3/12 | 17/48 | 12/45 | 0.842 | 0.710 |
| ADHD hyperactive | 2/12 | 10/48 | 5/45 | 0.461 | 0.316 |
| ADHD combined | 2/12 | 6/48 | 5/45 | 0.461 | 0.937 |
| ODD | 1/12 | 11/47 | 5/45 | 0.911 | 0.184 |
| CD | 1/12 | 9/47 | 1/45 | 0.284 | **0.045** |
| ODD or CD | 2/12 | 14/47 | 5/45 | 0.477 | 0.057 |
|  |  |  |  |  |  |

**Dichotomized symptom scale according to possible and certain symptoms (high/low dimensions of phenotype)

^*^Number of children with high dimensions of phenotype symptom/total number with the specific genetic marker

^†^SS and LL compared

^‡^LS and LL compared
