# Supplementary material for: ADHD and Disruptive behavior scores – associations with MAO-A and 5-HTT genes and with platelet MAO-B activity in adolescents
Source: BMC Psychiatry. 2008 Apr 23;8:28. doi: 10.1186/1471-244X-8-28 (PMC2383890; doi:10.1186/1471-244X-8-28)
Supplement: Additional file 4 — Dimensional symptom scales of ADHD and disruptive behavior related to genetic markers for 5-HTT LPR genotype in girls. [file 1471-244X-8-28-S4.doc]

**Additional file 4 - Dimensional symptom scales of ADHD and disruptive**

**behavior related to genetic markers for 5-HTT LPR genotype in girls**

|  |  | | |  |  |
| --- | --- | --- | --- | --- | --- |
|  | 5-HTT genotype in girls | | |  |  |
|  |  |  |  |  |  |
| Dimensional  symptom scale | SS  short/short  n^*^=27 | LS  long/short  n=56-57 | LL  long/long  n=56 | p^†^ | p^‡^ |
|  | Mean(S.D.) | Mean(S.D.) | Mean(S.D.) |  |  |
|  |  |  |  |  |  |
| ADHD inattentive | 2.78(2.19) | 3.09(3.77) | 3.59(3.98) | 0.079 | 0.409 |
| ADHD hyperactive | 3.96(2.08) | 2.80(3.23) | 2.48(2.74) | 0.088 | 0.863 |
| ADHD combined | 6.74(3.88) | 5.64(5.75) | 6.07(5.87) | 0.778 | 0.492 |
| ODD | 1.81(2.04) | 1.19(1.91) | 1.36(1.75) | 0.358 | 0.784 |
| CD | 0.52(1.05) | 0.42(1.05) | 0.79(1.45) | 0.376 | **0.045** |
| ODD or CD | 2.33(2.96) | 1.61(2.74) | 2.14(2.69) | 0.853 | 0.253 |
|  |  |  |  |  |  |

^*^Total number with the specific genetic marker

^†^SS and LL compared

^‡^LS and LL compared
